# Commentary: Top-down and bottom-up modulation of pain-induced oscillations

**DOI:** 10.3389/fnhum.2016.00152

**Published:** 2016-04-18

**Authors:** Valentina Nicolardi, Elia Valentini

**Affiliations:** ^1^Department of Psychology, Sapienza University of RomeRome, Italy; ^2^Fondazione Santa Lucia, Istituto di Ricovero e Cura a Carattere ScientificoRome, Italy; ^3^Department of Psychology and Centre for Brain Science, University of EssexEngland, UK

**Keywords:** pain, attention, gamma-band, EEG, top-down modulation

Attention represents one of the main mechanisms of modulation of pain (Wiech et al., 2008). It is a complex function which collects cognitive processes responsible for orienting to, and maintaining brain resources on, the most salient and relevant information for the ongoing behavioral demands (Knudsen, 2007). Whether attention can be captured by and oriented to sensory events depends on several variables, e.g., stimulus features, task demands, and volition (Baluch and Itti, 2011). Bottom-up capture of attention is often conceived as an unintentional and automatic process ruled by the competition between stimulus features, whereas top-down selection concerns an intentional process pertaining to intrinsic motivational and task-related aspects ruled by the subject (Buschman and Miller, 2007). Stimuli interpreted as painful are able to capture individual's attention in a bottom-up fashion if e.g., other stimuli or unrelated plans do not compete with its processing in working memory (Legrain et al., 2012).

In a recent issue of Frontiers in Human Neuroscience, Hauck et al. (2015) used electroencephalography (EEG) to investigate the oscillatory neuronal modulation exerted by both top-down and bottom-up attention during nociceptive painful stimulation. To disentangle how the two attentional processes influence neuronal synchronization and desynchronization, the authors manipulated both nociceptive stimulus intensity (i.e., high vs. low; bottom-up orientation) and spatial attention to the stimuli (i.e., attended vs. unattended; top-down control).

The authors already investigated the effect of spatial attention on cortical oscillatory activity elicited by electrical painful stimuli (Hauck et al., 2007) by recording magnetoencephalography (MEG) during an oddball paradigm whereby participants were asked to count the rare stimuli on one finger while ignoring the more frequent stimuli on the other finger. The authors concluded that increased attentional capture by salient rare stimuli was reflected by MEG-induced oscillatory high frequency activity (gamma band) localized in the sensory-motor areas. Hauck et al. (2015) expand on their previous methodology by varying location and intensity of laser painful stimuli. Here the participant had to attend and evaluate only stimuli delivered to one finger while ignoring those delivered to the other finger. At variance with their previous work, they delivered equiprobable stimuli on the ring and index finger, thus avoiding a bottom-up confounding effect associated with variable probability of occurrence of the stimuli.

They investigated attentional effects on the entire spectral activity and found that power (i.e., magnitude) of gamma oscillations increased (maximal at 270 ms post-stimulus) while alpha decreased (750 ms) during stimulation of the attended compared to the unattended finger. Conversely, the increase of stimulus intensity was associated with power increase in delta (300 ms) and gamma bands, and power decrease in alpha and beta bands (600 ms). Source analysis identified the contralateral insula as generator of gamma activity during top-down attentional control, and the sensory-motor and mid-cingulate regions as gamma activity generators during bottom-up capture of attention. The activation found in the sensory-motor areas during bottom-up attention is not surprising as previous studies reported the association between activity in the primary somatosensory cortex and selective nociceptive laser stimulation (Timmermann et al., 2001; Gross et al., 2007; Schulz et al., 2012; Valentini et al., 2012; Zhang et al., 2012).

A relevant aspect of Hauck et al.'s study (Hauck et al., 2015) is the differential modulation of gamma-band found during top-down and bottom-up attention to pain. To date, only Tiemann et al. (2015) explicitly investigated the functional significance of nociceptive-related gamma activity in these two conditions. They separately varied stimulus intensity, as a means to induce bottom-up modulation whereas they set out a placebo analgesia manipulation to induce the top-down modulation. Interestingly, while the design implemented by Tiemann et al. provides an orthogonal manipulation (i.e., intensity varies independently from cognitive expectation, and viceversa), the design devised by Hauck et al. did not manipulate the two variables separately, that is they required the participants to assess the intensity only for the stimuli delivered to the attended finger. Thus, they likely introduced a significant interaction between bottom-up and top-down processes in the “attended high” condition. Yet, their design does not allow to disambiguate this interaction.

Tiemann et al. (2015) found that nociceptive laser-evoked potentials were similarly influenced by both stimulus intensity and placebo analgesia whereas nociceptive-induced alpha and gamma responses were sensitive to changes in stimulus intensity but not to the induction of changes in beliefs about the experience of pain. Taking a closer look, one could argue that Hauck et al. (2015) may have enhanced the role of sensory features involved in the spatial localization of the stimuli compared to the placebo procedure implemented by Tiemann et al. (2015), which more consistently triggered higher-level cortical activity over the sensory cortices (Wager et al., 2011).

Other studies reported a gamma-band modulation by top-down attention during different sensory modalities (Fries et al., 2001). Using a top-down attentional manipulation similar to Hauck et al. (2015) and Siegel et al. (2008) characterized the effect of attention on neuronal synchronization pattern across the human dorsal visual pathway. During the post-stimulus interval they found higher gamma vs. lower alpha phase coherence between cortical regions involved in the processing of visual stimuli, and concluded that gamma band enhancement likely reflects local neuronal synchronization that is functional to the selective attention task. During subdural electrocorticography, Ray et al. (2008) investigated the effect of selectively attending one stimulus modality over the other in a crossmodal auditory and vibro-tactile stimulation. The authors found that gamma activity was greater over the auditory and somatosensory cortices when the auditory and tactile stimuli were attended. Overall, these studies support Hauck et al.'s (2015) interpretation that top-down selective attention is correlated with increased activity of gamma frequencies.

Despite differences in stimuli and methodology, altogether these studies indicate the gamma-band oscillatory activity may not only index bottom-up salience of the sensory event but also top-down relevance of the sensory representation for the ongoing behavioral goals. However, further methodological and analytical efforts will be needed to clarify the role of the gamma activity across different types of top-down attention manipulations.

## Author contributions

VN has developed most of the content of the article. EV has supervised her during the writing and he has edited the text.

### Conflict of interest statement

The authors declare that the research was conducted in the absence of any commercial or financial relationships that could be construed as a potential conflict of interest.
